# Supplementary material for: Content-rich biological network constructed by mining PubMed abstracts
Source: BMC Bioinformatics. 2004 Oct 8;5:147. doi: 10.1186/1471-2105-5-147 (PMC528731; doi:10.1186/1471-2105-5-147)
Supplement: Additional File 2 — The original results of the above study (non-essential files are deleted to keep the file size under the limit set by BMC bioinformatics). [file 1471-2105-5-147-S2.bz2 › chilibotAdditionalFile2/dip40/1ID9194558E1/html/BAX_BCL2L1.html]

 


 **BAX** and **BCL2L1** 
  
Found 1196 abstracts in PubMed, retrieved 40.  
 

 What does Google say? 
 PDF only 
| .edu only 

---

**Interactive relationship** (e.g. stimulation, inhibition, etc)

**Inhibitory relationship**- ... Bcl xl  [ **BCL2L1** ]  induction suppresses a p53 and  **Bax**  dependent apoptotic pathway.  Ref: 12855666 Clin Cancer Res, 2003
- It was reported that SCF c kit, Bcl 2 and Bcl xl  [ **BCL2L1** ]  inhibited the apoptosis while caspase 3, Fas,  **Bax**  and clusterine stimulated it.  Ref: 12861841 Zhonghua Nan Ke Xue, 2003
- A small inhibitor of the interaction between  **Bax**  and Bcl X  [ **BCL2L1** ]  L can synergize with methylprednisolone to induce apoptosis in Bcl X  [ **BCL2L1** ]  L overexpressing breast cancer cells.  Ref: 12884026 J Cancer Res Clin OncolJ Cancer Res Clin Oncol, 2003
- We have identified a small inhibitor of the interaction between  **Bax**  and Bcl X  [ **BCL2L1** ]  L that can synergize with methylprednisolone to induce apoptosis in Bcl X  [ **BCL2L1** ]  L overexpressing breast cancer cells.  Ref: 12884026 J Cancer Res Clin OncolJ Cancer Res Clin Oncol, 2003
**Neutral relationship**- Using an assay based on biosensor technology, we screened a chemical library of 1 00 compounds for inhibitors of the interaction between  **Bax**  and Bcl X  [ **BCL2L1** ]  L.  Ref: 12884026 J Cancer Res Clin OncolJ Cancer Res Clin Oncol, 2003
- To identify inhibitors of the interaction between  **Bax**  and Bcl X  [ **BCL2L1** ]  L.  Ref: 12884026 J Cancer Res Clin OncolJ Cancer Res Clin Oncol, 2003
- We further report that I3C upregulates  **Bax**  Bcl 2 ratio and downregulates Bcl xL  [ **BCL2L1** ]  expression in CA1a cells but not in MCF10A cells.  Ref: 12791510 Nutr Cancer< MedlineTA>Nutr Cancer, 2003

**Non-interactive relationship** (e.g. studied together, co-existance, homology, etc.)

- These results demonstrate that the mitogenic and anti apoptotic effects of insulin on bovine preimplantation embryos did not correlate with changes in the amounts of mRNA for the glucose transporter isoforms Glut and 8, or transcripts for  **Bax**  and Bcl X  [ **BCL2L1** ]  L.  Ref: 12814351 Reproduction< MedlineTA>Reproduction, 2003
- **Bax**  was detected in homogenate and cytosol, and its level was not affected by ethanol.CONCLUSIONS DLPC, at a dose contained in PPC, reproduces the antiapoptotic actions of PPC through a reduction in cytosolic cytochrome c concentration and caspase 3 activity, possibly in association with up regulation of Bcl x  [ **BCL2L1** ]  L expression.  Ref: 12824822 Alcohol Clin Exp Res, 2003
- **Bax**  and Bcl xL  [ **BCL2L1** ]  proteins were not changed, although Bcl 2 proteins increased with troglitazone.  Ref: 12840598 Am J Nephrol< MedlineTA>Am J Nephrol, 2003
- CONCLUSIONS Upregulation of Bcl 2 and Bcl xl  [ **BCL2L1** ]  but not  **Bax**  may have a role in protective ischemic preconditioning.  Ref: 12791942 Stroke< MedlineTA>Stroke, 2003
- **Bax** , a death promoting protein, was upregulated and Bcl x  [ **BCL2L1** ]  L, a death inhibiting protein, was downregulated.  Ref: 12830326 Eur J Nucl Med Mol Imaging< MedlineTA>Eur J Nucl Med Mol Imaging, 2003
- The  **Bax**  Bcl xl  [ **BCL2L1** ]  ratio changed little.  Ref: 12855666 Clin Cancer Res, 2003
- Here, we found that HDB induced apoptotic cell death was accompanied by upregulation of cyclin D3,  **Bax** , and p21 and down regulation of Bcl X  [ **BCL2L1** ]  L, while HDB had no effect on the levels of Bcl 2 and Bad protein.  Ref: 12822933 J Agric Food Chem< MedlineTA>J Agric Food Chem, 2003
- We also analyzed the relationship between TCF 4 gene splicing isoforms, proliferation proliferating cell nuclear antigen labeling index, and apoptosis antiapoptotic factors Bcl 2 and Bcl x  [ **BCL2L1** ]  L, proapoptotic factors Bak and  **Bax** , and caspase 3 in RCC samples.  Ref: 12796377 Clin Cancer Res< MedlineTA>Clin Cancer Res, 2003
- iii the frequency of apoptosis in blastocysts was determined by the TdT mediated duTP nick end labelling TUNEL assay and by quantification of the relative amounts of mRNA for  **Bax**  and Bcl X  [ **BCL2L1** ]  L.  Ref: 12814351 Reproduction< MedlineTA>Reproduction, 2003
- Survivin expression did not correlate with bcl 2, bcl x  [ **BCL2L1** ] , or  **bax**  expression, stage, or overall or disease free survival.  Ref: 12808063 Mod Pathol, 2003
- We assessed survivin expression in ovarian carcinoma, correlating results with expression of other anti apoptotic bcl 2, bcl x  [ **BCL2L1** ] , mutant p53 and pro apoptotic  **bax**  markers, with prognostic parameters, and prognosis.  Ref: 12808063 Mod Pathol, 2003
- PURPOSEWe hypothesized that apoptosis is a downstream event in erectile dysfunction, and pro apoptotic Bak and  **Bax**  and anti apoptotic Bcl 2 and Bcl x  [ **BCL2L1** ]  factors are involved in the etiology of diabetes induced erectile dysfunction.  Ref: 12796708 J Urol< MedlineTA>J Urol, 2003
- METHODS The expressions of apoptosis associated genes bcl 2,  **bax** , bcl X  [ **BCL2L1** ]  L and bcl X  [ **BCL2L1** ]  S and the activity of caspase 3 were studied by reverse transcription polymerase chain reaction RT PCR and western blot in the cisplatin resistant A2780 DDP, COC1 DDP and sensitive human ovarian cancer cells A2780 and COC1.  Ref: 12816691 Zhonghua Fu Chan Ke Za Zhi, 2003
- Although changes in the expression of apoptosis related proteins Bcl 2, Bcl xl  [ **BCL2L1** ] , and  **Bax**  have been considered to be crucially important in ischemic injury, the roles these proteins play in ischemic preconditioning induced by sublethal forebrain ischemia have not been elucidated.  Ref: 12791942 Stroke< MedlineTA>Stroke, 2003
- Expression of  **BAX**  and Bcl XL  [ **BCL2L1** ]  were probed in the cells treated with or without Erbitux.Results.  Ref: 12850474 J Surg Res, 2003
- Remarkably, these events occurred in the absence of any reduction in the expression of the Bcl 2 family members Bcl 2, Mcl 1 and Bcl XL  [ **BCL2L1** ] , or any change in the proapoptotic molecules Bad or  **Bax** .  Ref: 12842827 Am J Physiol Gastrointest Liver Physiol< MedlineTA>Am J Physiol Gastrointest Liver Physiol, 2003
- In these conditions, microscopic analysis revealed chromatin condensation in dying cells and the Bcl 2, Bcl x  [ **BCL2L1** ]  L  **Bax**  mRNA balance was altered.  Ref: 12832039 FEBS Lett, 2003
- iii analysis of  **Bax**  and Bcl XL  [ **BCL2L1** ]  gene expression by RT PCR.  Ref: 12813566 Cell Mol Biol Lett, 2003
- ... levels of other members of the Bcl 2 family, such as  **Bax**  and Bcl x  [ **BCL2L1** ] , did not change in paclitaxel resistant cell lines.  Ref: 12815160 Mol Pharmacol< MedlineTA>Mol Pharmacol, 2003
- SN38 induced p53,  **Bax** , Bcl xl  [ **BCL2L1** ] , and p53 dependent p21 WAF1 protein accumulation.  Ref: 12855666 Clin Cancer Res, 2003
- The expressions of Fas and Bcl 2 family genes  **Bax** , Bcl 2, Bcl xL  [ **BCL2L1** ] , Bcl xS  [ **BCL2L1** ]  were not affected by NS 398 treatment in all three cell lines.  Ref: 12792783 Int J Oncol, 2003
- The protein contents of cytochrome c, procaspase 3, caspase 3, Bcl x  [ **BCL2L1** ]  L, and  **Bax**  were analyzed by Western blot and quantified by densitometry.  Ref: 12824822 Alcohol Clin Exp Res, 2003
- Experimental Design Using wild type HCT116, p53 null,  **Bax**  null, or p21 WAF1 null isogenic derivatives, we measured expression of regulators of cellular response, and associated growth arrests or apoptosis, after SN38 treatment, with or without antisense mediated Bcl xl  [ **BCL2L1** ]  knockdown.  Ref: 12855666 Clin Cancer Res, 2003
- Induction of Apoptosis by Hydroxydibenzoylmethane through Coordinative Modulation of Cyclin D3, Bcl X  [ **BCL2L1** ]  L, and  **Bax** , Release of Cytochrome c, and Sequential Activation of Caspases in Human Colorectal Carcinoma Cells.  Ref: 12822933 J Agric Food Chem< MedlineTA>J Agric Food Chem, 2003
- AIMS To correlate the expression of a series of apoptotic and oncogene markers including p53, Bcl 2,  **BAX** , Bcl XL  [ **BCL2L1** ] , p21WAF, 1 CIP1, cyclin D1, HER 2 neu in thymic epithelial tumours with histological type, stage and resectability and to determine whether the information on HER 2 neu would be valuable in identifying patients who are eligible for anti HER 2 neu treatment.  Ref: 12877732 Histopathology, 2003
- In wild type HCT116, but not in  **Bax**  null cells, Bcl xl  [ **BCL2L1** ]  knockdown induced a shift in response from drug induced senescence to apoptosis, and enhanced the global cytotoxicity of SN38.  Ref: 12855666 Clin Cancer Res, 2003
- Moreover, Bcl 2 and Bcl xL  [ **BCL2L1** ]  protect neurons from apoptosis, while  **Bax**  and Bcl xS  [ **BCL2L1** ]  may act as proapototic proteins.  Ref: 12820987 Neurochem Int, 2003
- METHODS We performed Northern blot analysis for  **bax** , bcl x  [ **BCL2L1** ]  L and bcl 2 at both temperatures.  Ref: 12824868 Eur J Endocrinol, 2003
- Simvastatin appears to alter the balance between cell life and death promoting genes, as reflected by the decreased Bcl xL  [ **BCL2L1** ]   **Bax**  ratio.  Ref: 12787408 Kidney Int< MedlineTA>Kidney Int, 2003
- Different Expression Patterns of Bcl 2, Bcl xl  [ **BCL2L1** ] , and  **Bax**  Proteins After Sublethal Forebrain Ischemia in C57Black Crj6 Mouse Striatum.  Ref: 12791942 Stroke< MedlineTA>Stroke, 2003
- ... other Bcl 2 family proteins including  **Bax** , Bcl 2, Bcl XL  [ **BCL2L1** ] , and Bag remained unchanged.  Ref: 12874837 J Cell Biochem, 2003
- No effects of insulin on the mRNA expression of Glut isoforms and  **Bax**  and Bcl X  [ **BCL2L1** ]  L were found.  Ref: 12814351 Reproduction< MedlineTA>Reproduction, 2003
- Paraffin embedded sections of 49 ovarian carcinoma were immunostained for survivin, bcl 2, bcl x  [ **BCL2L1** ] ,  **bax** , and p53.  Ref: 12808063 Mod Pathol, 2003
- RESULTS Ribonuclease protection assay data indicate an age related increase in  **Bax** , Bcl xL  [ **BCL2L1** ] , and procaspase 3 messenger RNA expression in aged compared with young rats.  Ref: 12862407 Am J RhinolAm J Rhinol, 2003
- Frequency of survivin, bcl 2, bcl x  [ **BCL2L1** ] ,  **bax** , and p53 was 73.5%, 36.7%, 93.9%, 77.6%, and 60.4%, respectively.  Ref: 12808063 Mod Pathol, 2003
- Therefore, we investigated the transcription and expression of Bcl 2, Bcl xl  [ **BCL2L1** ] , and  **Bax**  in striatum of mice subjected to sublethal forebrain ischemia and lethal ischemia, with or without ischemic preconditioning.  Ref: 12791942 Stroke< MedlineTA>Stroke, 2003
- No change was detected in Bcl 2,  **Bax** , or Bcl xL  [ **BCL2L1** ]  proteins.  Ref: 12830043 J Thorac Cardiovasc Surg, 2003
- There was no difference found in the expression of proapoptotic  **Bax**  and Bcl X  [ **BCL2L1** ]  s or antiapoptotic Bcl 2 and Bcl X  [ **BCL2L1** ]  L genes nor in the expression of the tumor suppressor gene p53.  Ref: 12885947 J Leukoc Biol, 2003
- Immunoprecipitation studies revealed that Nbk interacts with Bcl x  [ **BCL2L1** ]  L and Bcl 2 but not with  **Bax** .  Ref: 12853473 EMBO J< MedlineTA>EMBO J, 2003
